# STarMirDB: A database of microRNA binding sites

**DOI:** 10.1080/15476286.2016.1182279

**Published:** 2016-05-04

**Authors:** William Rennie, Shaveta Kanoria, Chaochun Liu, Bibekanand Mallick, Dang Long, Adam Wolenc, C. Steven Carmack, Jun Lu, Ye Ding

**Affiliations:** Wadsworth Center, New York State Department of Health, Center for Medical Science, Albany, NY, USA

**Keywords:** Binding site prediction, CLIP, microRNA, seed site, seedless site

## Abstract

microRNAs (miRNAs) are an abundant class of small endogenous non-coding RNAs (ncRNAs) of ∼22 nucleotides (nts) in length. These small regulatory molecules are involved in diverse developmental, physiological and pathological processes. miRNAs target mRNAs (mRNAs) for translational repression and/or mRNA degradation. Predictions of miRNA binding sites facilitate experimental validation of miRNA targets. Models developed with data from CLIP studies have been used for predictions of miRNA binding sites in the whole transcriptomes of human, mouse and worm. The prediction results have been assembled into STarMirDB, a new database of miRNA binding sites available at http://sfold.wadsworth.org/starmirDB.php. STarMirDB can be searched by miRNAs or mRNAs separately or in combination. The search results are categorized into seed and seedless sites in 3′ UTR, CDS and 5′ UTR. For each predicted site, STarMirDB provides a comprehensive list of sequence, thermodynamic and target structural features that are known to influence miRNA: target interaction. A high resolution PDF diagram of the conformation of the miRNA:target hybrid is also available for visualization and publication. The results of a database search are available through both an interactive viewer and downloadable text files.

## Introduction

miRNAs are a class of single-stranded, non-coding RNAs of ∼22 nucleotides in length. They have been discovered in plants, animals as well as in some viruses.^1-3^ miRNAs play essential roles in cell proliferation, differentiation, development, and are associated with human diseases.^2,4^ A mature miRNA can guide miRNA-induced silencing complex (miRISC) for target recognition by sequence complementarity between the miRNA and sequences typically in the 3′ untranslated regions (3′ UTRs) of the cognitive mRNAs (mRNAs). Successful target binding usually results in translational repression and/or mRNA degradation.^5^ Each human miRNA is predicted to be able to regulate several hundred different mRNAs.^6^

Computational prediction algorithms have proven to be valuable in the discovery of new miRNA targets. Most of the existing algorithms are based on the seed rule, i.e., the target site within 3′ UTR forms Watson-Crick (WC) pairs with bases at positions 2 through 7/8 of the 5′ end of the miRNA.^7^ However, numerous exceptions to the seed rule have been well-documented.^8-13^ Other sequence features have been proposed based on their enhancement of targeting specificity. These include sequence conservation, strong base-pairing to the 3′ end of the miRNA, local AU content and location of miRNA binding sites (near either end of the 3′ UTR is favorable).^14^ The importance of target structural accessibility for miRNA target recognition has been supported by numerous studies.^15-21^

In recent years, experimental methods based on cross-linking immunoprecipitation (CLIP) have been developed. For human and mouse studies, these include HITS-CLIP,^22^ PAR-CLIP^23^ and variations of such techniques.^24^ The CLIP approach has also been successful in worm.^25^

The CLIP studies have provided high throughput quality datasets for regions of mRNAs containing miRNA binding sites. These data allowed us to develop models for improved predictions of miRNA binding sites.^18,26^ The models are based on a comprehensive list of sequence, thermodynamic and target structure features that were enriched for miRNA binding sites identified from CLIP data, and were validated by intra-data set, inter-dataset as well as cross-species validations. For human, mouse and worm, we have used these models to carry out transcriptome-scale predictions of both seed and seedless sites in the 3′ untranslated region (3′ UTR), coding sequence (CDS) region, and 5′ untranslated region (5′ UTR) of mRNAs. The results have been assembled into STarMirDB, a new database application module of the Sfold RNA package.^27,28^ In this article, we describe this new resource. The unique tools of STarMirDB shall complement the existing miRNA target resources for computational predictions and experimental target data. Examples of these include, but are not limited to, TargetScan,^29^ Diana-microT,^30^ TarBase,^31^ StarBase,^32^ miRecords^33^ and miRTarBase.^34^

## Generation of transcriptome-scale data for STarMirDB

The database currently contains records for 3 species, *H. sapiens* (human), *M. musculus* (mouse) and *C. elegans* (worm). For human and mouse, we used complete mRNA sequences from NCBI RefSeq build 36.3 and 37.2, respectively. For worm, 3′ UTR sequences were obtained from the Wormbase version WS-190. The current release of STarMirDB includes 38,745 transcripts for human, 34,631 for mouse and 22,926 3′ UTRs for worm. miRNA sequences were obtained from miRBase release 18.^35^ We collected 1,921 miRNA sequences for human, 1,157 for mouse and 368 for worm.

Our CLIP based models were used to make transcriptome scale predictions of both seed and seedless binding sites.^18^ For each site, a comprehensive list of sequence, thermodynamic and target structure features are computed (Table 1). A logistic probability is provided as a measure of confidence in the predicted site. The number of binding sites is astronomical, so that in the database we only included those with a probability of 0.5 or higher. This filter also helps assure a reasonable response time for database search queries. In the case of interest in those low confidence sites with probabilities under 0.5, the user can use the STarMir web server that presents all predicted sites for single or multiple miRNAs and a target mRNA.^36^ The database can be searched by one or more miRNAs or targets, separately or in a combination. For worm, we provide a user interface that allows developmental stage specific search of miRNA binding sites within the 3′ UTR of transcripts. This interface is activated when *C. elegans* is selected as the species for database search. Additionally, for *C. elegans*, all the prediction data for miRNA binding sites within the 3′ UTR of transcripts are also provided as downloadable files.

**Table 1.** Description of site information and features for STarMirDB output.

Site ID	Predicted sites are sequentially numbered along the target sequence
Target	Accession number of the target mRNA
Gene	Gene symbol of the target mRNA
miRNA	Name of the microRNA (miRNA)
Target_Len	Length of the target
Site_Position	Start and end position of the target region (site) predicted to be bound by miRNA
Seed_Position	Start and end position of the target sub-region complementary to the miRNA seed (i.e. positions 2–7/8 of the miRNA)
Seed_Type	6mer, offset 6mer, 7mer-A1, 7mer-m8, and 8mer seed sites ^14, 45^
Site_Access	A measure of structural accessibility as computed by the average probability of a nucleotide being single-stranded (i.e., unpaired) for the nucleotides in the predicted binding site^18^
Seed_Access	A measure of structural accessibility as computed by the average of single-stranded probabilities of the nucleotides in the target sub-region complementary to the miRNA seed^18^
Upstream_Access (# nt)	A measure of structural accessibility as computed by the average of single-stranded probabilities for the block of nucleotides upstream of the predicted binding site (# nt: block size of 5, 10, 20, 25 or 30)^18^
Dwstream_Access (# nt)	A measure of structural accessibility as computed by the average of single-stranded probabilities for the block of nucleotides downstream of the predicted binding site^18^
Upstream_AU (# nt)	Percentage of AU for the block of nucleotides upstream of the binding site
Dwstream_AU (# nt)	Percentage of AU for the block of nucleotides downstream of the binding site
Site_Location	Relative starting location of the predicted binding site along the length of the sequence(e.g., for 3′ UTR, 0 indicates the 5′ end of the UTR, and 1 corresponds to the 3′ end)^14^
3′_bp	Presence of contiguous Watson Crick base pairing for miRNA nucleotide positions 12–17 (sites with 3′_bp are also called 3′ compensatory/supplementary sites)^14^
Site_Consv	Conservation score by the PhastCons program^46^ for the binding site
Seed_Consv	Conservation score by the PhastCons program for the target sub-region complementary to the miRNA seed
Offseed_Consv	Conservation score by the PhastCons program for nucleotides within the target site, but outside the seed complementary region
dG_hybrid	ΔG_hybrid_ (in kcal/mol): a measure of stability for miRNA:target hybrid as computed by RNAhybrid^45^
dG_nucl	ΔG_nucl_(in kcal/mol): a measure of the potential of nucleation for miRNA:target hybridization^17^
dG_total	ΔG_total_(in kcal/mol): A measure of the total energy change of the hybridization^17^
LogitProb	Logistic probability of the site being an miRNA binding site as predicted by our logistic model^18^
Target_Mismatch	Nucleotides in the target binding site that are not base paired with the miRNA
Target_Match	Nucleotides in the target binding site that are base paired with the miRNA
Mir_Match	Nucleotides in the miRNA that are base paired with the target mRNA
Mir_Mismatch	Nucleotides in the miRNA that are not base paired with the target mRNA
Hybrid Conformation	The last 4 fields above present information for the miRNA:target hybrid conformation predicted by RNAhybrid. In each of the fields, spaces are included so the fields can be easily aligned to produce a simple diagram of the hybrid conformation as illustrated below: Target_Mismatch: U UUUCC U A Target_Match: GACU AUGUA CUACCUC Mir_Match: UUGA UACGU GAUGGAG Mir_Mismatch: UGGAU A

## Input of database query

STarMirDB presents a collection of predicted miRNA binding sites on mRNAs through a web interface that enables both search and retrieval of data and visualization of the conformation of predicted miRNA:target hybrid for each predicted site. The web interface has four input fields: species, miRNAs, mRNAs, and logistic probability threshold. The requirements for each input field are described below in detail.

To start the database search, the user should first select the species from a dropdown menu. Currently three species are included in the database: human (*Homo sapiens*), mouse (*Mus musculus*) and worm (*C. elegans*). Next, one or a set of miRNAs can be selected from the miRNA scroll down list, which displays all available miRNAs assembled for the selected species. Additionally, one or more miRNA names can be entered in the text box. The database follows the naming convention used by miRBase,^35^ i.e., all the miRNAs can be identified by their miRNA name/identifier (e.g., *hsa-let7-5p* for human, *mmu-let7a* for mouse, and *cel-mir-1018* for worm).

Target mRNA information has to be entered into the provided text box. For human and mouse, either Genbank accession number or Gene symbol, as assigned by the HUGO Gene Nomenclature Committee (HGNC), can be provided. For worm, Wormbase ID is required. For search result display through an interactive site viewer, a user can choose to display only the most relevant site features for each binding site, or the complete list of features. The most relevant features are considered by us to be the most informative. They were selected from those used in the development of the prediction models.^18,26^ A user may choose to input merely miRNAs while leaving the target input box blank. In this case, the database server will retrieve predicted sites for the entire transcriptome assembled for the species. The user can also choose to input merely mRNA IDs, which will prompt the database server to identify all miRNAs assembled for the species that have binding sites on those mRNAs. This can be useful, e.g., when the question is whether an mRNA is targeted by any miRNA. A database search is typically instantaneous. However, if the database is queried with only miRNA(s) without target information, the search will take minutes. Finally, the user can use a drop down menu to filter out miRNA binding sites with logistic probabilities below the specified threshold.

## Output of database query

Relevant data in the database are retrieved in response to a specific database query and are available through both an interactive site viewer and downloadable files. For the interactive site viewer, the data is classified into three mRNA regions (5′ UTR, CDS and 3′ UTR) and seed and seedless sites. To facilitate online viewing, the number of sites displayed in the interactive area is limited to top-ranked sites according to the decreasing order of their logistic probabilities. By default, 100 binding sites are displayed. Alternatively, the user can choose to display the top 250, 500 or 1,000 sites. The results of a search are also available for download as text files, wherein all of the retrieved binding sites are listed. The interactive viewer presents the results with either the most relevant site features or all of the site features as specified by the user in the input page. The downloadable text files provide all site features. In the text files, features marked with an asterisk are those used in the model computations of the logistic probabilities. In addition to comprehensive sequence, thermodynamic and target structural features (Table 1), a high resolution PDF diagram of the conformation of the miRNA:target hybrid is also provided. The diagram was developed to be high quality so that the user can choose to use them for publication purposes. When both the miRNA and the mRNA were included in the CLIP study for the prediction model development,^18,26^ an indicator field named “CLIP” will be given a value of 1 if the predicted site is supported by the CLIP data, and 0 otherwise. CLIP studies are limited to abundant miRNAs and expressed transcripts. When either the miRNA or the mRNA was absent in the CLIP study, a value of “NA” is assigned to the CLIP indicator. In the database, less than 1% of sites have a CLIP indicator value of 0 or 1. Thus, our prediction data complement the CLIP data. A file providing definitions of site features is available via the link for ‘Feature definitions’ under the table listing predicted sites.

## Illustration of database search

**Figure 1.**
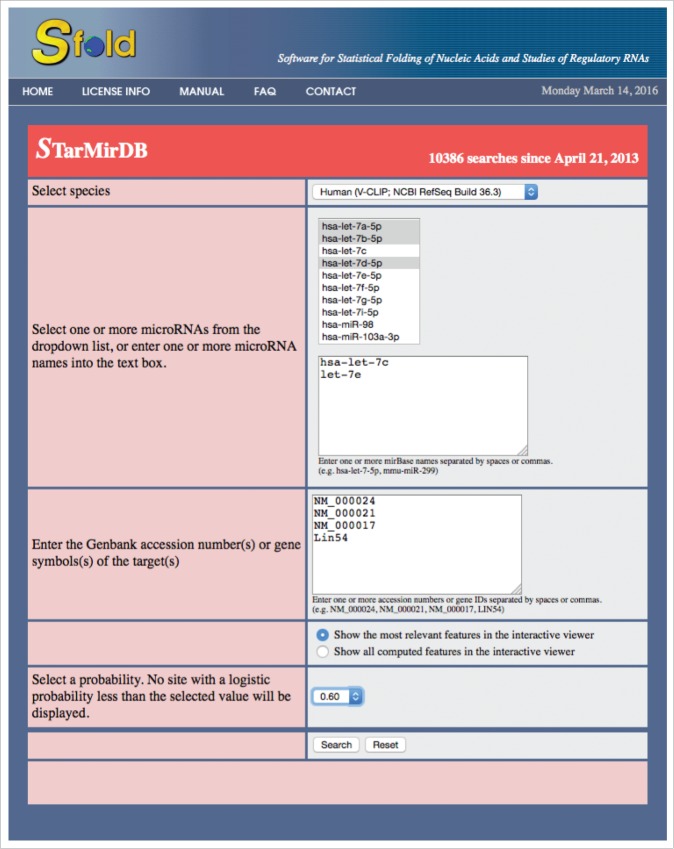
An illustrative example of the STarMirDB input page. Binding sites are searched for miRNAs selected from a pre-stored list as well as manually entered by a user, multiple targets, and a specific logistic probability threshold selected by the user. The option of the most relevant site features is selected for output display.

For an illustration of the database search, Fig. 1 shows the input screen for a query starting with ‘Human (V-CLIP; NCBI RefSeq Build 36.3)’ selected in the species dropdown menu. From the dropdown list of miRNAs, *hsa-7a-5p, hsa-7b-5p*, and *hsa-7d-5p* were selected. In addition, 2 miRNAs, *hsa-let-7c* and *hsa-let-7e* were manually entered. For mRNA targets, accession numbers NM_0000024, NM_0000021, NM_0000017, and Gene symbol *Lin54* were entered. Next, the option of “Show predictions with the most relevant features in interactive viewer” was selected. Finally, a logistic probability threshold of 0.6 was selected. The “Search” button was then clicked for submitting the query input information for processing by the database server.

**Figure 2.**
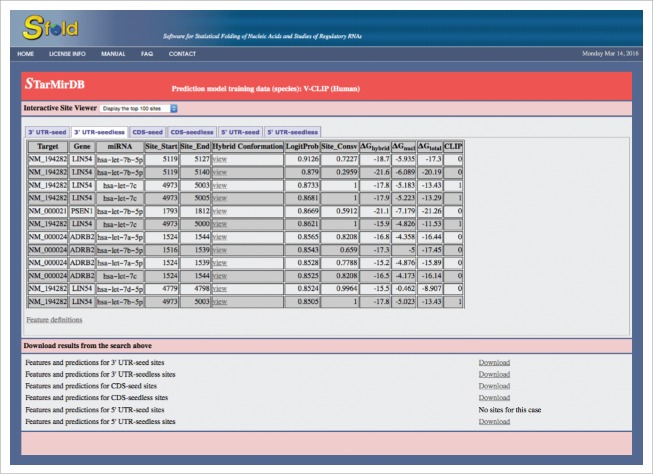
STarMirDB output page for the default display of top 100 sites, with the tab selected for displaying seedless sites in the 3′ UTR.

**Figure 3.**
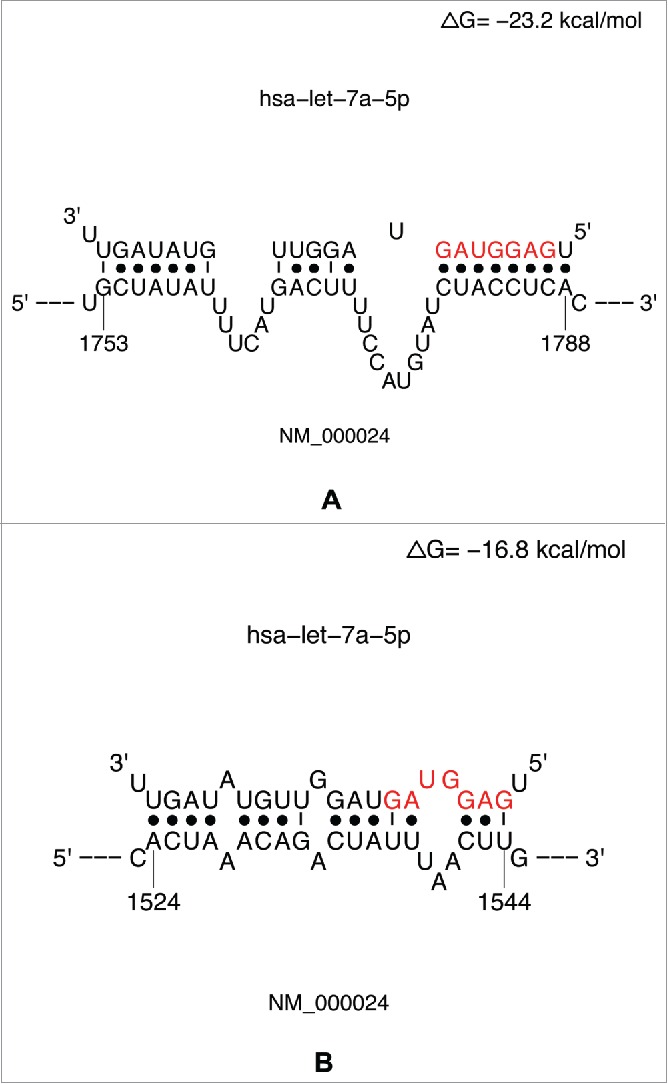
Conformation diagrams of miRNA:target hybrids for a seed site (A) and a seedless site (B).

Upon completion of data retrieval by the database server, the user is presented with an interactive site viewer (Fig. 2). By default, the list of the top 100 sites is displayed in decreasing order of logistic probabilities. An alternative number of sites can be selected from a dropdown menu. The tab for “3′ UTR-seedless” was selected for presenting seedless sites in the 3′ UTR of the target. For example, the first entry in the site table has a logistic probability of 0.9126, which indicates a high confidence in this predicted site. A rather low value of −17.3 kcal/mol for ΔG_total_ indicates a high structural accessibility at the target site.^17^ In the “Hybrid Conformation” column, a link is provided for a high resolution PDF diagram of the conformation of the miRNA:target hybrid at the predicted site. Clicking this link will open the diagram in a new tab or window, depending on the configuration of the user's web browser. Multiple windows/tabs facilitate comparison of hybrid conformations for multiple binding sites. Fig. 3 shows hybrid diagrams for a seed site and a seedless site.

Under the interactive site viewer, links are provided for downloading files of the query results for the 6 combinations of regions and site types (Fig. 2). For site feature information, the downloadable files provide all site features whereas the interactive viewer displays either all or the most relevant features as selected by the user in the query input page. The user can initiate a new search by clicking on the link at the bottom of the page.

## Conclusions

STarMirDB is a new bioinformatics resource for facilitating miRNA target studies. The current release of database includes 96,302 mRNAs and 3,446 miRNAs for human, mouse and worm. It will be periodically updated and likely extended to other species. It presents predictions for all 3 mRNA regions and for both seed and seedless sites. Importantly, it presents a probability for each site as an indicator of confidence in the prediction. In addition to use for visualization and publication, high quality diagrams of miRNA:target hybrids can facilitate design of nucleotide mutations for experimental validation of binding sites. The option for search by developmental stage shall be useful for studies of miRNAs in worm. The unique tools from STarMirDB will complement the existing miRNA target resources for computational predictions and experimental target data. The database can retrieve miRNA binding sites for single or multiple miRNAs and/or one or more targets. For example, this capability will be useful for elucidating miRNA regulation of genes of interest. It will also be useful in miRNA overexpression and knockout studies, wherein differentially expressed genes can be further examined by prediction and validation of miRNA binding sites.

We have also developed STarMir, a web server for prediction of miRNA binding sites.^36^ STarMir and STarMirDB are complementary tools. While the database allows fast search of pre-computed results, STarMir makes predictions for any miRNA:mRNA pair from any species of interest. For example, the user can use STarMir in making predictions for a new isoform absent in the current database release.

The provision of extensive predictions of seedless sites (i.e., non-canonical sites) is a major feature for both the database and the web server. The functionality of seedless sites has been demonstrated by numerous studies based on diverse methods, which include reporter assays, nucleotide mutation analysis, analysis of microarray data, analysis of proteomics data, and phenotypic analysis.^11,37-43^ However, a study primarily based on microarray data failed to find support for functional seedless sites.^44^ Further experimental investigations will be helpful for addressing this lack of consensus. Our tools will facilitate experimental testing of predicted seedless sites, especially those with high logistic probabilities.
